# Is Lockdown Bad for Social Anxiety in COVID-19 Regions?: A National Study in The SOR Perspective

**DOI:** 10.3390/ijerph17124561

**Published:** 2020-06-24

**Authors:** Lei Zheng, Miao Miao, JiYoon Lim, Maorui Li, Shu Nie, Xiaojun Zhang

**Affiliations:** 1 School of Economics and Management, Fuzhou University, Fuzhou 350108, China; leizh@fzu.edu.cn (L.Z.); 071907110@fzu.edu.cn (M.L.); 2 Center for China Social Trust Research, Fuzhou University, Fuzhou 350108, China; 3 Institute of Psychological and Cognitive Sciences, Fuzhou University, Fuzhou 350108, China; 4 School of Society, China University of Political Science and Law, Beijing 102249, China; miaomiao@cupl.edu.cn; 5 School of Psychological and Cognitive Sciences and Beijing Key Laboratory of Behavior and Mental Health, Peking University, Beijing 100871, China; 1901110653@pku.edu.cn (J.L.); 1801110669@pku.edu.cn (S.N.); 6 Institute for Risk and Disaster Reduction, University College London, London WC1E 6BT, UK

**Keywords:** lockdown measures, pandemic COVID-19, anxiety, SOR model

## Abstract

Lockdown measures have been widely used to control and prevent virus transmission in pandemic regions. However, the psychological effects of lockdown measures have been neglected, and the related theoretical research lags behind the practice. The present study aimed to better understand the mechanism of social anxiety in pandemic regions where the lockdown measures were imposed, based on the conceptual framework of the Stimulus-Organism-Response (SOR). For that, this research investigated how lockdown measures and psychological distance influenced social anxiety in the pandemic region. The Chinese national data was analyzed for the outcome. The results showed that (1) psychological distance mediated the relationship between pandemic COVID-19 severity and social anxiety, (2) lockdown measures buffered the detrimental effect of the COVID-19 pandemic severity on social anxiety, (3) lockdown measures moderated the mediation effect of psychological distancing on social anxiety caused by the COVID-19 pandemic. In conclusion, under the SOR framework, the lockdown measures had a buffer effect on social anxiety in pandemic regions, with the mediating role of psychological distancing.

## 1. Introduction

The new corona virus, or COVID-19, has become a pandemic with accelerating incidence. Since this disease has been confirmed to have interhuman transmission, the Chinese government actively adopted lockdown measures to cut off the channels of infection for virus prevention and control. The crisis governance measures include ensuring resident registration before people leave their houses, as well as the closing down of the roads, railways, and public transports. Consequently, lockdown measures have been adopted by several countries and regions [1,2]. In late February, the Italian government took lockdown measures to cope with the spread of the virus in some North Italian areas [3].

To date, the measures exhibited their effect on infection prevention and control, showing an obvious decrease in coronavirus cases in mainland China. However, as a crisis governance measure, the widespread lockdown inevitably imposed psychological effects on social emotions [4], particularly anxiety. In fact, existing studies have demonstrated the increase in social anxiety in pandemic areas [5,6,7], which might lead to riots, violence [8,9,10]. So, do these crisis governance measures really have an impact on social emotions in pandemic regions? What are the mechanisms underlying this process?

Within the theory of the Stimulus-Organism-Response (SOR), the present study addressed the psychological effect of lockdown measures in a pandemic area. From the perspective of psychological distancing, the lockdown measures restricted traffic and outdoor activities. By that, the individuals’ perceived spatial distancing toward the disease increased, viewing the disease as a high-level construal [11]. Conversely, measures such as movement restriction prolonged home-stay, which subsequently leaded to anxiety and depression [12]. Not only the psychological effect of lockdown measures regarding responses toward COVID-19 remains unclear, but also there is no current study investigating the mechanisms underlying certain processes. 

Therefore, this study will add empirical evidence regarding the psychological effect of the lockdown measures from the governmental level to the individual-level. Importantly, crisis governance measures could be an approach to buffer the effect of a pandemic on social emotions. Also, although numerous studies have confirmed the detrimental effects of pandemic diseases on anxiety, there is no study that revealed the mechanisms underlying this process. Based on the SOR theory, this study will propose a conceptual model to explain how citizens evaluate a pandemic from the perspective of psychological distancing and why lockdown buffers the social anxiety caused by COVID-19.

## 2. Conceptual Model and Hypothesis

**Figure 1:**
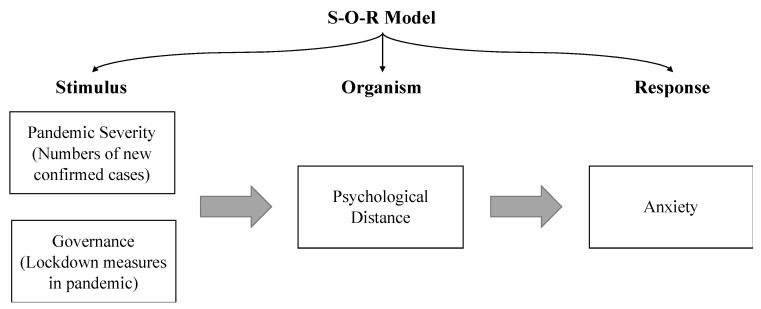
Conceptual model.

The Stimulus-Organism-Response or SOR framework is a cognitive theory for predicting and identifying individuals’ reactions, including emotion. In the SOR framework, environmental factors could affect individuals’ reactions (e.g., anxiety) through the organismic variable (e.g., psychological distance) [13,14]. This model offers a visualized framework to study the pandemic severity and lockdown as environmental stimuli on social anxiety and reveals the mechanism underlying that specific process. Based on the SOR model, we thus proposed an overarching framework to explain the mechanism of how crisis governance impacts social anxiety caused by COVID-19 in ways of psychological distance (Figure 1). 

### 2.1. Pandemic Severity and Social Anxiety

Threats, such as the rapidly spreading virus, often lead people to feel discomfort and tension [15,16]. In fact, pandemic diseases were found to be associated with high-level anxiety. For example, the epidemic of Severe Acute Respiratory Syndrome (SARS) caused anxiety and social problems in several countries [5,6,7]. Similarly, the influenza H1N1, also known as “Swine Flu”, resulted in considerable public anxiety in 2009, especially after WHO declared it as a pandemic [17]. A Singapore study also demonstrated the detrimental effect of “Swine Flu” on public anxiety, in which people reported high level of anxiety [18]. In line with that, another UK study confirmed that people who read or received the government’s Swine Flu leaflet reported high levels of anxiety [6]. 

These studies provide evidence on public anxiety caused by pandemics. In fact, the feelings of anxiety are highly associated with individuals’ perceived severity [19]. Subsequently, people may perceive high levels of infection risk when there is an increasing number of COVID-19 new cases nearby, thus resulting in increased social anxiety. Therefore, we hypothesize that:

**Hypothesis 1:**  *Pandemic severity positively predicts social anxiety in pandemic regions.*

### 2.2. Psychological Distance and Social Anxiety

Although numerous studies tested social anxiety caused by pandemics, the mechanisms underlying certain processes is still unclear. Previous studies suggested that the perceived distance toward the disease had an impact on subjects’ emotions [11,20]. Psychological distancing is the subjective perception between self and others, such as people, future events, or possibility. Therefore, the individuals’ perceived psychological distanceing between the disease and themselves affect their psychological outcomes, such as anxiety. 

This is in line with the construal level theory [21,22], which claims that people develop different psychological representations of a disease based on their perceived mental distance towards it [11]. In terms of the far psychological distance, people tend to view objects as in ways of high-level construal. Conversely, for the close psychological distancing, a low-level construal is employed as the concept with concrete, specific, and contextualized features [20,21]. Therefore, when people perceive a high infectious risk toward a disease, their psychological distance decreases, which, in turn, leads to a low-level construal.

The existing research found that distance between people and the affected place could predict the levels of anxiety caused by an infectious virus [7,23]. In particular, medical students at the affected hospital reported higher anxiety than non-medical students of the same university [7]. Comparably, students at another university situated 20 km away from the affected hospital reported the lowest level of anxiety.

When people perceived the virus at a remote distance, they tend to construe the virus at a high level with less emotional responses, thus exhibiting lower levels of anxiety. Conversely, when people perceived the virus at a nearby distance, they tend to be irrational with more emotional responses, which in turn exhibit higher levels of anxiety. Given the different numbers of new COVID-19 cases across provinces in China, people might become more anxious if their province reports a comparatively larger number of new COVID-19 cases than other provinces. The reason might be that individuals perceive a larger number of new cases in nearby provinces as a higher possibility of being infected by COVID-19. Therefore, we propose that:

**Hypothesis 2:**  *Psychological distance serves as a mediator to explain the relationship between pandemic severity and social anxiety in pandemic regions.*

### 2.3. Lockdown, Psychological Distance, and Anxiety

Lockdown measures have been adopted to contain infectious diseases in previous outbreaks. In 2015, the Sierra Leone government ordered its citizens to remain indoors for three days for virus prevention and control. These measures were thought to have played an important role in the Ebola elimination [24,25,26,27]. In China, there are two main aspects of lockdown measures in the prevention and control of COVID-19; “traffic control” and “household quarantine advice”. Since the local governments in China held different policies, the extent of lockdown measures also differed among provinces. 

Although the lockdown showed its impact on COVID-19 control [19,28,29], its psychological effect is still unclear. The literature is very sparse in terms of the relationship between lockdown measures and social anxiety. In fact, people are intrinsically motivated to flee when they face threats, which also results in anxiety [30]. One research has demonstrated that prolonged homestays contribute to anxiety and depression [12]. Therefore, people might have perceived an anxious feeling due to the movement restriction after lockdown. Consequently, people in provinces with a high level of lockdown measures may report higher levels of anxiety than people under low levels of lockdown measures. In this perspective, lockdown may increase the anxiety level of people in provinces under a serious COVID-19 situation. Thus, we hypothesize that:

**Hypothesis 3a:**  *People in provinces with high-level lockdown measures exhibit more anxiety compared to people who are under low-level lockdown measures.*

**Hypothesis 3b:**  *Lockdown strengthens the negative effect of pandemic severity on social anxiety in pandemic regions.*

On the other hand, people may feel less anxious when lockdown cuts off virus transmission and reduces the possibility of infection. The research found that traffic control is an effective way to cut off the virus transmission, limiting the spread of infection [31,32]. Studies also demonstrated that household quarantine significantly limits the spread of infectious disease [33,34,35]. After lockdown measures, the possibility of COVID-19 infection will be reduced due to the decreased interpersonal interaction. Therefore, lockdown measures may buffer the detrimental effect of the pandemic severity, resulting in the reduction of social anxiety. Thus, we propose that:

**Hypothesis 4a:**  *Lockdown buffers the effect of the pandemic severity, reducing anxiety.*

Todorov et al. (2007) proposed that infection probability also changes the individuals’ psychological distance. Therefore, when the infection possibility is perceived as low, people might show an increase in subjective perception toward spatial distancing regarding COVID-19. In other words, the far distance would lower the anxiety [20,35]. Thus, since lockdown measures cut off the interpersonal contact, people in provinces with high-level lockdown measures may perceive COVID-19 as a remote disease, which may protect them from anxiety. Thus, we propose that:

**Hypothesis 4b:**  *The lockdown moderates the mediation effect of psychological distancing; therefore, reducing the effect of the severity of the pandemic on anxiety.*

## 3. Materials and Methods

### 3.1. Participants

There were 1847 participants enrolled in the present study. Their age ranged from 18–66 years. The mean age is 30.64 ± 9.19 years. Seven hundred sixty-seven of them were males. Their demographic information is represented in Table 1. The research protocol was approved by the Ethics Committee of Fuzhou University. All participants were recruited by a widely used social platform (i.e., WeChat) by online fliers, which direct them to the online survey website. Each of them was first informed about the research online and they submitted the informed consent before starting the research. All participants completed the survey within 10 min on 7 February 2020.

### 3.2. Measures

The psychological distancing was assessed by two items with statements “please indicate how far do you feel a sense of distance between you and the coronavirus” and “please indicate how far do you feel a sense of distance between you and the confirmed people” [20]. Each item was responded to using a five-point scale (1—extremely near, 9—extremely remote). The Cronbach’s alpha was 0.90.

Anxiety was measured by a short version of the State Anxiety Scale, which has been widely used in previous research [36]. The scale included six items, such as calm, tense, upset, relaxed, content, and worry. Each item was responded to using a seven-point scale ranging from 1—never to 7—always. The Cronbach’s alpha was 0.73.

The severity of the pandemic was measured by the number of newly confirmed COVID-19 cases. The data were collected by the National Health Commission of the People’s Republic of China. The present study used the data on the day the survey was taken. 

The extent of lockdown measures was measured using the traffic control index collected from the Baidu migration big data database(qianxi.baidu.com). We collected big data of migration for 14 days from “Chunjie” (Chinese Lunar New Year) to the day before the survey was taken for both 2019 and 2020. The extent of the lockdown measures was evaluated by comparing the averaged migration index difference between 2019–2020. A high difference value indicated that the local government adopted comparably strict lockdown measures within the province.

## 4. Results

### 4.1. Descriptive Information

Table 1 shows statistics regarding demographics, pandemic severity, lockdown, psychological distancing, and anxiety. According to the correlations, the severity of the pandemic had a negative correlation with psychological distancing (*r* = −0.14, *p* < 0.001), and a positive correlation with anxiety (*r* = 0.07, *p =* 0.004); lockdown had a negative correlation with anxiety (*r* = −0.10, *p* < 0.001), and was positively associated to psychological distancing (*r* = 0.10, *p* < 0.001). Psychological distancing had a negative correlation with anxiety (*r* = −0.17, *p* < 0.001).

### 4.2. Pandemic Severity Impacts Anxiety Through Psychological Distancing

This study conducted a Hierarchical Linear Model, in which regions as a cluster, psychological distancing, and anxiety were taken as within-level variables, and pandemic severity as the between-level variable. 

This study employed Model 1 with anxiety as the dependent variable and demographic variables as control variables. Next, the pandemic severity was added in Model 2. Subsequently, psychological distancing was included with either a fixed effect (model 3) and random effect (model 4), respectively. Last, a mediation model was developed to estimate the mediating effect of psychological distance between pandemic severity and anxiety in model 5.

As shown in Table 2, the results of model 1 showed that females reported higher levels of anxiety compared to males (B = 0.24, SE = 0.05, *p* < 0.001). Similarly, highly educated people reported higher anxiety than low-educated people (B = 0.07, SE = 0.03, *p =* 0.03). However, neither age nor monthly income significantly predicted anxiety in the presented sample. 

Furthermore, in model 2, pandemic severity positively predicted anxiety (B = 0.07, SE = 0.01, *p* < 0.001, △R^2^ = 0.04), indicating that people in the provinces with a larger number of new cases perceived a higher level of anxiety, which is consistent with H1. 

In model 3, we found that psychological distancing negatively predicted anxiety (B = −0.17, SE = 0.02, *p* < 0.001, △R^2^ = 0.03) by a fixed-effect approach. After conducting a random effect model, psychological distancing consistently predicted anxiety (B = −0.17, SE = 0.03, *p* < 0.001, △R^2^ = 0.03) in model 4. Lastly, in model 5a and 5b, we found that psychological distancing served as a mediator, explaining the route from pandemic severity at the province-level to anxiety at the individual-level (Random Effect Size = 0.02, SE = 0.004, *p* < 0.001). As H2 hypothesized, the results suggested that the pandemic severity impacted anxiety through psychological distancing. 

### 4.3. Lockdown Buffers Pandemic by Moderating Psychological Distance Against Anxiety 

In order to test the moderation effect of the lockdown, we first conducted model 6 with lockdown as the predictor. Next, the interaction item of the lockdown and pandemic severity was included in model 7. Subsequently, we developed model 8 with psychological distance as the dependent variable, and lockdown and pandemic severity as independent variables. Further, an interaction item of lockdown and pandemic severity was included in model 9. Last, a cross-level moderated mediation effect of lockdown was conducted (model 10). 

As shown in Table 3, pandemic severity consistently predicted anxiety (B = 0.04, SE = 0.01, *p =* 0.003) in model 6. Moreover, lockdown also negatively predicted anxiety (B = −0.08, SE = 0.04, *p =* 0.056), indicating that lockdown reduced social anxiety in pandemic regions. Therefore, our results support H4 instead of H3.

**Figure 2:**
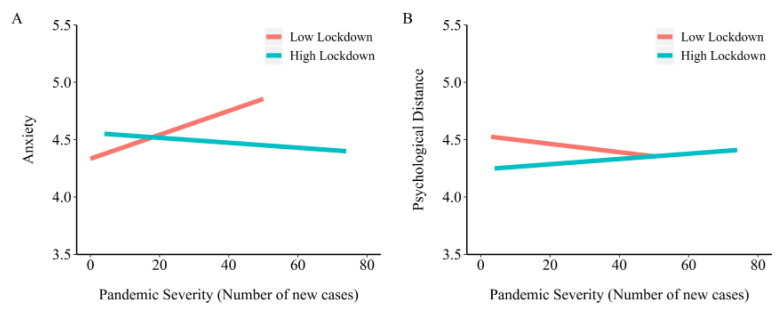
The moderation effect of lockdown on psychological outcomes. (**A**) indicates the moderating effect of lockdown on the relationship between pandemic severity and anxiety; (**B**) indicates the moderating effect of lockdown on the relationship between pandemic severity and psychological distance.

In model 7, lockdown interacted with pandemic severity to predict anxiety (B = −0.11, SE = 0.05, *p =* 0.025, △R^2^ = 0.001). Subsequently, the results of a simple slope analysis showed that pandemic severity positively predicted anxiety in provinces with low levels of lockdown (B = 0.08, SE = 0.39, *p =* 0.036) than that in provinces with high levels of lockdown (B = −0.23, SE = 0.14, *p =* 0.09; Figure 2A). 

In addition, lockdown consistently predicted psychological distancing in model 8 (B = 0.08, SE = 0.04, *p =* 0.037). Subsequently, the result also demonstrated the mediation effect of psychological distancing on the relationship between lockdown and anxiety (Random Effect Size = −0.013, SE = 0.007, *p =* 0.024). Furthermore, the interaction of lockdown and pandemic severity positively predicted psychological distancing (B = 0.10, SE = 0.04, *p =* 0.027). 

The results of a simple slope analysis showed the negative prediction of the pandemic severity on psychological distancing in provinces with low levels of lockdown (B = −0.08, SE = 0.04, *p =* 0.038) compared to provinces with high levels of lockdown (B = 0.05, SE = 0.09, *p =* 0.555; Figure 2B).

Lastly, the results of the cross-level moderated mediation model showed that lockdown buffered the detrimental effect of pandemic severity on anxiety through psychological distancing regulations. Specifically, the mediation effect of psychological distancing on the relationship between pandemic severity and anxiety was not significant (B = −0.01, SE = 0.02, *p =* 0.557) in provinces with high-level lockdown, but was significant in provinces with low-level lockdown (B = 0.02, SE = 0.01, *p =* 0.047).

## 5. Discussion

Based on the SOR theory, the present study developed a conceptual model, in which lockdown moderated the detrimental effect of pandemic severity on social anxiety. In addition, the results also demonstrated the mediation effect of psychological distancing underlying the process between environmental factors (e.g., lockdown and pandemic severity) and social anxiety in the pandemic region.

### 5.1. The SOR Model Explains How Pandemic Impacts Emotions

Although the existing research demonstrated the detrimental effect of the pandemic on individuals’ mental health, the mechanism underlying this process was still unclear. Given the theory of the SOR model, environmental factors could affect individuals’ reactions (e.g., anxiety) through the organismic variable (e.g., psychological distance) [13,14]. Our results provide evidence that both pandemic severity and lockdown serve as environmental factors at the regional level, which changed the perception of psychological distancing at a personal level, thus increasing the individuals’ anxiety. This supported and extended the SOR model in a pandemic study, in which environmental variables come from the regional level, and organismic and response variables come from individual levels.

In addition, environmental factors could also interact to affect individuals’ psychological variables. The pandemic has been regarded as a detrimental factor that reduces the psychological distance toward COVID-19, thus negatively predicting anxiety. However, as discussed before, lockdown reduced psychological distancing, which, in turn, relieved anxiety [11,20,36]. Importantly, our findings suggested that the crisis governance measures (e.g., lockdown) serves as an environmental factor that can buffer the effect of other environmental factors (e.g., pandemic), thus affecting on the individuals’ psychological variables. Therefore, crisis governance measures could play an important role in both pandemic control and the public’s mental health. 

### 5.2. Evidence for the Psychological Effects of Lockdown in the Pandemic Region

This study confirmed the association between regional pandemic intensity and individuals’ anxiety in China, which is consistent with other results regarding pandemic and anxiety [19]. Moreover, psychological distancing was proven to serve as a mediator in the relationship between pandemic severity and social anxiety. It is consistent with previous studies, which reported that people’s psychological distance toward disease impacts their responses related to the disease [7,11]. Not only that, but it also explains the mechanism of how the pandemic affects social emotions.

It is noteworthy that the present sample provided empirical evidence of the psychological effect of lockdown measures using a national sample. The result showed that lockdown not only negatively predicted individuals’ anxiety but also buffered social anxieties against the detrimental impacts of the pandemic. Given the differential governance across provinces in China, we found that lockdown in intensive pandemic regions reduced individuals’ anxiety levels. 

More importantly, our findings suggested the buffer effect of lockdown in pandemic regions. In China, people residing in lockdown regions were required to stay at home, which increased social distancing and reduced the possibility of infection. As mentioned before, when the possibility decreased, psychological distancing increased [20]. Consistent with that, we confirmed that lockdown predicted low levels of anxiety in ways of increasing psychological distancing. This suggested that the crisis governance measures could affect people’s cognition, and consequently, their emotions. Therefore, the lockdown measure served as a critical function for protecting people against the biological and psychological virus. Hence, the measures tended to increase psychological distancing towards COVID-19, which helped people cope with the threat. 

Altogether, our findings on lockdown and psychological distancing not only provided direct evidence about whether lockdown has psychological effects in pandemic regions or not, but it also carried important implications for understanding why lockdown buffered social anxiety caused by COVID-19.

### 5.3. Social Psychological Problems in Epidemic Governance

Social psychology plays an important role in epidemic governance. From the social psychology perspective, the direct causation of behavior is the primary function of emotion [37,38], people in a strong state of psychological stress will make a variety of irrational behavior, and even coupled with the impact of events to cause secondary disasters. The present study built a conceptual model based on the SOR model, in which lockdown interacted with pandemic severity to predict psychological distancing, and thereby result in social anxiety. 

In addition, the psychological effects of the lockdown play an important role in crisis governance. Our results revealed that pandemic severity negatively predicted anxiety in provinces with low levels of lockdown compared to the provinces with high levels of lockdown. Therefore, when we take the lockdown measures, region-specific situations and environmental risks should be considered. In the society under risk, the crisis governance measures could also induce other potential risks, namely the risk of operation failure, regardless of whether the society is risk or safety-oriented. Specifically, the government’s crisis governance (i.e., “institutionalization” of risk) also could become a new risk to the society (i.e., “institutionalized” risk) [39]. Consequently, there might be other psychological effects that could hurt social stability, especially in other countries. Therefore, when we take action to contain the pandemic risk, social psychology should be taken seriously to avoid the risk of human judgmental errors.

### 5.4. Practical Implications

In response to major public health events, a positive social mentality helps work together to overcome the epidemic. The breeding and spreading of negative emotion, conversely, posed severe challenges to social governance in public emergencies. Our research discovered the interaction between lockdown and pandemic severity on social anxiety through psychological distancing. This result offers several practical implications for both governors and citizens. 

The lockdown could be an effective measure to slow down the virus spread; however, people’s lives are greatly affected by this measure. So, governors should realize that it could also be an extreme measure that could trigger social anxiety, where the emotional changes of individuals and groups should not be neglected, as it may lead to successive economic and social problems. Considering the psychological mechanism, governors should take measures to reduce psychological distancing, collecting, and publishing accurate information about pandemic severity, enhancing personal prevention and control measures, and looking into cognitive and emotional changes of individuals and groups at each node based on the main laws regarding the evolution of emergencies. Furthermore, for crisis governance, emergency living and medical supplies should not be neglected before lockdown. The citizens need to stay calm, learn about the infectious disease, obey the government guidelines, and take self-protection procedures at home.

### 5.5. Limitations

The current research has several limitations. First, this study employed a cross-sectional design. Although the sample size for this research is relatively large, it is impossible to determine the causal inference, as well as whether people under lockdown measures in the long-term show similar results. Moreover, the participants finished their online survey and were compensated after the study. This may lead to sampling bias due to internet access issues. Besides, their internal motivation and emotion may be affected by the compensation of this study. Further longitudinal studies should be conducted as a replication of this research to address this issue.

Second, the present study evaluated psychological variables relying solely on self-reported measurements. Although these inventories are widely used in previous studies, self-reported measurements may run the risk of responses being influenced by participants’ moods. 

Third, even though lockdown is a complicated procedure, this research only considered two main aspects of lockdown—“traffic control” and “household quarantine advice”—in China. In fact, the lockdown measures include several aspects, which were also conducted differently across countries. In particular, lockdown measures are the compulsory policy in most countries, but not in all countries. For example, Daegu, the fourth largest city of Korea, has been declared as a “special management zone” due to the outbreak of corona virus. However, the Korean government did not lockdown the city or citizens, except for the facilities that were related to the outbreak [40]. There should be a difference between the two policies in terms of their psychological effects. Therefore, other aspects of the lockdown procedure need to be examined and fulfilled in future operations.

Forth, since China is a collectivistic society, the cultural aspect might have played a factor that impacted the relationship between lockdown and anxiety, as well as the moderating effect of lockdown. This may restrict the generalization of the findings in this paper. Therefore, future studies should consider cultural aspects of the psychological effects during the lockdown procedure, and repeat our research in other countries with different cultural backgrounds.

## 6. Conclusions

In conclusion, this study provided evidence that the lockdown measures buffered anxiety against the detrimental effect of COVID-19. More importantly, psychological distancing explained why a pandemic impacts people’s anxiety and how lockdown moderates the relationship between pandemic severity and anxiety. In addition, considering that some countries are starting to adopt similar measures, this study suggests to consider the psychological effects of crisis governance measures in pandemic regions. Given that anxiety is often caused by uncertainty, an objective description of the pandemic by the public government may minimize the negative psychological effects of the lockdown policy.

## Figures and Tables

**Table 1:** Demographic and variables information.

Variable	Mean/Categories	Total No.
Age	30.64 ± 9.19	1847
Gender	Males	767
	Females	1080
Education	High school	257
	Junior college	465
	Bachelor	868
	Master or PhD	257
Income	<1999	423
	2000–3999	430
	4000–5999	423
	6000–9999	332
	10,000–14,999	156
	15,000–19,999	40
	>20,000	43
Psychological Distance	4.43 ± 2.03	
Anxiety	4.41 ± 1.05	
Pandemic Severity	174.15 ± 563.68	
Lockdown	3.71 ± 1.76	

**Table 2:** Cross-level mediation analysis for pandemic severity, psychological distancing, and anxiety.

Variables	Model 1	Model 2	Model 3	Model 4	Model 5*a*	Model 5*b*
	Anxiety	Anxiety	Anxiety	Anxiety	Anxiety	PD
**Sex**	0.23 ** (0.04)	0.23 ** (0.05)	0.22 ** (0.04)	0.22 ** (0.05)	0.22 ** (0.04)	−0.06 (0.06)
**Age**	0.01 (0.01)	0.01 (0.01)	0.01 (0.01)	0.01 (0.01)	0.01 (0.01)	0.01 (0.01)
**Education**	0.08 ** (0.03)	0.08 ** (0.03)	0.06 * (0.03)	0.06 * (0.03)	0.06 * (0.03)	−0.11 ** (0.03)
**Income**	−0.01 (0.02)	−0.01 (0.02)	−0.01 (0.02)	−0.01 (0.02)	−0.01 (0.02)	0.01 (0.01)
**PS**		0.06 (0.01)	0.04 ** (0.01)	0.04 (0.02)	0.04 (0.02)	−0.12 ** (0.01)
**PD**			−0.17 ** (0.02)	−0.17 ** (0.02)	−0.17 ** (0.02)	
						
**△R^2^**	0.02	0.003	0.03	0.03	0.03	0.03

Note: *—*p* < 0.05; **—*p* < 0.01; PD—Psychological Distance; PS—Pandemic severity was measured by the number of new confirmed cases at the province-level; Sex: 1—male, 2—female; Education: 1—high school, 2—college; 3—bachelor; 4—master or PhD; Monthly income: 1—below ¥1999; 2—2000–¥3999; 3—4000–¥5999; 4—6000–¥9999; 5—10,000–¥14,999; 6—15,000–¥19,999; 7—over ¥20,000.

**Table 3:** Cross-level moderated mediation analysis of lock down, pandemic severity, psychological distancing, and anxiety.

Variables	Model 6	Model 7	Model 8	Model 9	Model 10*a*	Model 10*b*
	Anxiety	Anxiety	PD	PD	Anxiety	PD
**Sex**	0.20 ** (0.04)	0.23 ** (0.05)	−0.01 ** (0.05)	−0.01 ** (0.05)	0.20 ** (0.05)	−0.01 (0.05)
**Age**	0.01 (0.01)	0.01 (0.01)	−0.01 (0.01)	−0.01 (0.01)	0.01 (0.01)	−0.01 (0.01)
**Education**	0.07 * (0.03)	0.08 ** (0.03)	−0.10 (0.03)	−0.10 (0.03)	0.05 (0.03)	−0.10 ** (0.03)
**Income**	−0.03 (0.02)	−0.01 (0.02)	0.01 ** (0.02)	0.01 ** (0.02)	−0.02 (0.02)	0.01 (0.02)
**PD**					−0.18 ** (0.03)	
**Pandemic Severity**	0.04 ** (0.01)	−0.08 (0.07)	−0.12 (0.04)	−0.02 (0.06)	0.06 (0.04)	−0.02 (0.06)
**Lockdown**	−0.08 + (0.04)	−0.11 (0.05)	0.08 ** (0.04)	0.10 ** (0.04)		0.10 ** (0.04)
**PS × LD**		−0.11 * (0.05)		0.10 ** (0.04)		0.10 ** (0.04)
						
**△R^2^**	0.06	0.001	0.08	0.001	0.02	0.001

Note: +—*p* < 0.10; *—*p* < 0.05; **—*p* < 0.01; PD—Psychological Distance; PS—Pandemic Severity, which is measured by the number of new confirmed cases at the province-level; Lockdown was measured by the traffic control data; LD—Lockdown; PS × LD was the interaction of pandemic severity and lockdown; Sex: 1—male, 2—female; Education: 1—high school; 2—college; 3—bachelor; 4—master or Ph.D; Monthly income: 1—below ¥1999, 2—2000–¥3999; 3—4000–¥5999, 4—6000–¥9999, 5—10,000–¥14,999, 6—15,000–¥19,999, 7—over ¥20,000.
